# Circadian Disruption Impacts Fetal Development in Mice Using High-Frequency Ultrasound

**DOI:** 10.5334/jcr.249

**Published:** 2024-12-20

**Authors:** Samantha M. Britz, Shay Nelson, Kylie M. Earhart, James K. Pru, Emily E. Schmitt

**Affiliations:** 1 WWAMI Medical Education, University of Washington School of Medicine, Seattle, WA, US; 2 Division of Kinesiology & Health, University of Wyoming, Laramie, WY, US; 3 Program in Reproductive Biology, Department of Animal Science, University of Wyoming, Laramie, WY, US

**Keywords:** circadian disruption, development, embryo, fetus, pregnancy, shift work

## Abstract

The developmental origins of health and disease theory suggests that environmental exposures during early life, particularly during prenatal life, can greatly influence health status later in life. Irregular light-dark cycles, such as those experienced during shift work, result in the repeated disruption of circadian rhythms, which negatively impacts physiological and behavioral cycles. The purpose of our study was to assess parameters in the developing mouse embryo and fetus using high frequency ultrasound when exposed to circadian disruption. Pregnant female mice were subjected to a seven-hour advanced circadian disrupted protocol or remained on a normal 12/12 light-dark cycle throughout pregnancy. Significant differences were observed in placental length (p = 0.00016), placental thickness (p = 0.0332), and stomach diameter (p = 0.0186) at E14.5–18.5. These findings suggest that circadian disruption in pregnant dams, mimicking shift work, alters embryonic and fetal development in specific organs *in utero*.

## Introduction

It is now well-established that environmental exposures during prenatal and early postnatal life can influence health status later in life. Specifically, this theory links environmental conditions during germ cell exposure, fetal development, and early life phases to an increased likelihood of metabolic disorders in adulthood [1]. For example, research shows that nutritional challenges during pregnancy and lactation periods could lead to an imbalance of energy regulation later in life contributing to the development of such disease [2]. Additionally, epidemiologic studies have correlated higher birth weights with increased chances of developing breast cancer [3].

Studies in female fertility clearly demonstrate that overall health leads to healthy reproductive tissues. Yet, an estimated 12–28% of working women are on shift work schedules or work over 48 hours per week [4]. There is increasing evidence that sleep quality and duration are important for female reproduction [5] and disruption of the circadian rhythms can lead to many deleterious health outcomes in the female reproductive system including: polycystic ovary syndrome [6,7,8], endometriosis [9], preeclampsia [10,11], pre-term birth [12], miscarriage [13,14], reproductive cancers [15,16], and even infertility [17,18]. The link between circadian disruption and female fertility remains poorly described, and underlying mechanisms essential for regulating circadian timing in the ovary and uterus need to be examined further.

The circadian system is responsible for many physiological functions, including body temperature, heart rate, hormone secretion, and regulation of the sleep-wake-cycle and is coordinated in a 24-hr rhythmic pattern. Irregular cycles of light-dark often seen in shift work results in the repeated reset of circadian rhythms, which disrupts physiological and behavioral cycles [19]. A central molecular clock known as the hypothalamic suprachiasmatic nucleus (SCN) acts as the pacemaker to multiple circadian oscillators in peripheral tissues, including those of the female reproductive tract. A core feedback loop is responsible for regulating the circadian system through heterodimeric transcription factors that cause the transcription of its inhibitors. This core feedback loop is regulated by the activator genes Clock (Circadian Locomotor Output Cycles Kaput) and Bmal1 (Brain and Muscle ARNT[Aryl hydrocarbon receptor nuclear translocator-like protein]-Like1) and regulate a number of accessory genes in the molecular clock pathway [20]. Such accessory genes include: Period1 (*PER1*), Period2 (*PER2*), Period3 (*PER3*), Cryptochrome1 (*CRY1*), and Cryptochrome2 (*CRY2*) which then feedback to suppress the Bmal/Clock heterodimer [21]. The regulation of clock proteins is controlled post-translationally by protein kinases, specifically casein kinase 1 delta (*CK1δ*) and epsilon (*CK1ɛ*) [22,23,24]. Ovulation is thought to be controlled by the molecular clock in mammalian follicular cells coordinating with peripheral circadian clocks in the ovary and uterus to further regulate the female reproductive system [25]. Additionally, deletion of BMAL1 causes failure of embryo implantation in the female knockout mice suggesting that peripheral clocks play a specialized role in reproductive biology [26]. Lastly, melatonin availability is crucial in both placental function and ovarian physiology, and exposure to light at night suppresses elevated melatonin levels, which is likely to negatively impact these systems through the disruption of the master circadian clock [19].

The data that exists on fetal circadian disruption centers mostly around negative outcomes that manifest later in life like behavioral issues [27,28] and risk of chronic disease [29] and not necessarily the fetal developmental growth during pregnancy. Little information is known about the impact of circadian disruption on maternal tissues versus fetal tissues, and the mechanisms by which fetal growth parameters could be directly or indirectly impacted by maternal circumstances remain unclear. Few, if any studies, have utilized high frequency ultrasound (HFUS) to measure anatomical parameters of the embryo, fetus, and placenta. Therefore, the purpose of this study was to assess growth parameters in the developing mouse embryo and fetus during pregnancy using HFUS when exposed to circadian disruption. Since past studies have associated disrupted circadian rhythms with preterm birth and low birth weights [30], we expect a number of measured anatomical measurements to be decreased in circadian disrupted mice.

## Methods

### Animals

**Figure 1:**
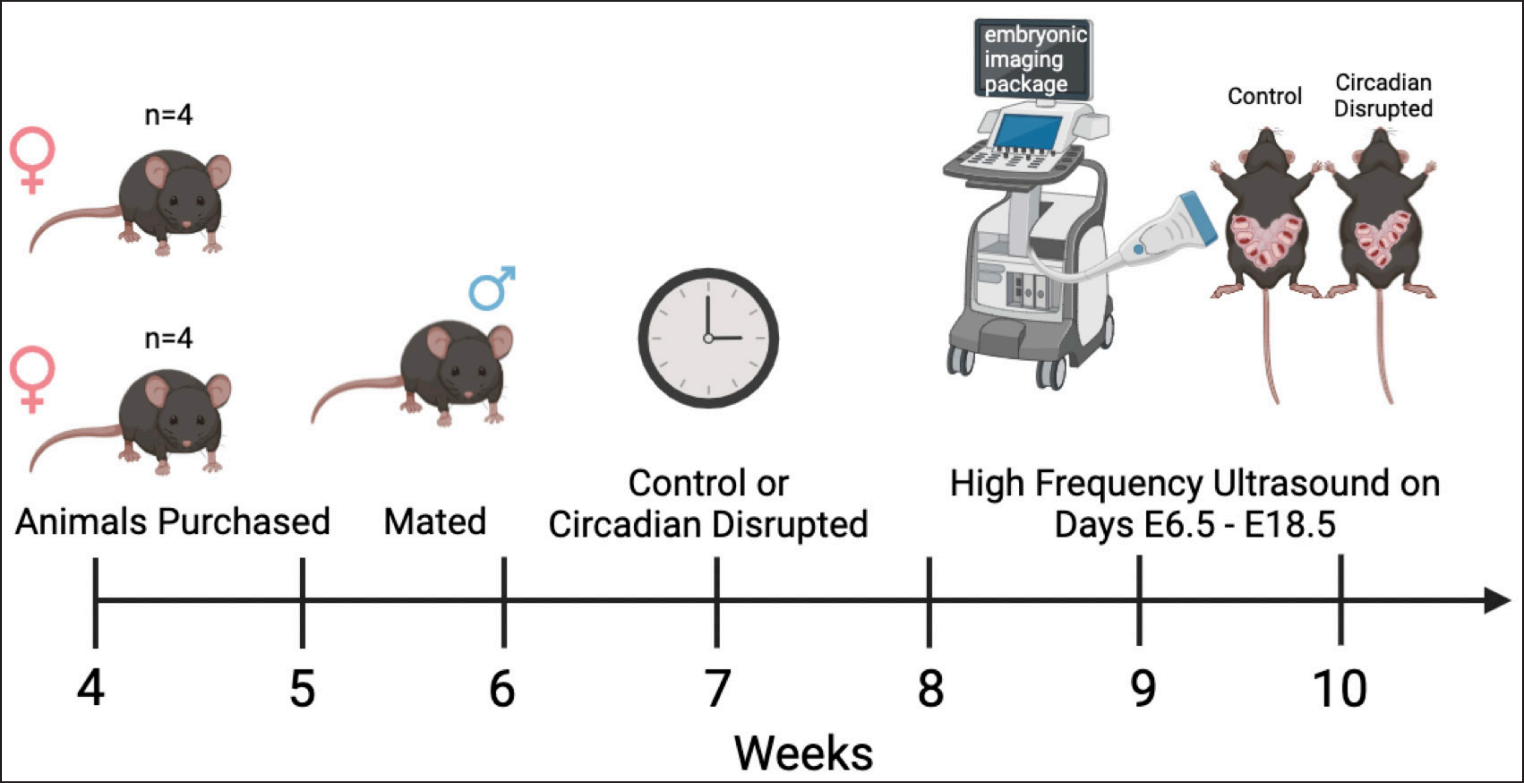
Experimental timeline.

All experiments and methods described in this study were conducted in accordance with institutional guidelines and approved by the Institutional Animal Care Users Committee at the University of Wyoming. Four-week-old virgin C57BL/6 J female mice were obtained from Jackson Laboratories (Bar Harbor, ME). Upon arrival, mice were housed in groups of 2 or 3 animals per cage and quarantined for two weeks and kept under a standard 12-hr light/dark cycle with lights on at zeitgeber [31] 0 and lights off at ZT12. Water and food were provided ad libitum. Breeding was then set up where 6-week old female mice were mated with 8-week old male mice and monitored for the presence of a vaginal plug. Once pregnancy was established, males were removed from the cage, n = 7 pregnant females were subjected to our circadian disruption protocol (7 hr light advance to induce circadian disruption) and n = 7 pregnant females remained as controls (normal 12/12 light-dark cycle) throughout the duration of pregnancy. The experimental timeline is illustrated in Figure 1.

### High Frequency Ultrasound (HFUS)

Embryos were imaged through the maternal abdominal wall using the embryonic imaging package on the VisualSonics Vevo 2100 (Fujifilm) HFUS to monitor fetal development in control and circadian disrupted female mice at the same time every day. We aimed to keep the measurements at a constant time daily (9–11AM). A total of 58 embryonic measurements (circadian disrupted n = 34, control n = 24) were recorded throughout the course of the study. HFUS was used to track fetal measurements on the animals over the course of pregnancy on days 6.5–18.5. Measurements included decidual thickness, gestational sac thickness and length, placental thickness and length, crown-rump length, occipital-snout length, and stomach diameter. Because it is not possible to measure each measurement parameter for the entire duration of the pregnancy, we divided the measurements into three time groups. The first set of parameters were measured on embryonic days 6.5–9.5. The second set of parameters were measured on embryonic days 10.5–13.5. The final measurements were recorded on days 14.5–18.5. All measurements were taken by the same technician to ensure consistency during the experiment. Measurements were recorded for each pregnancy and analyzed using the ultrasound software package, with reference to Greco et al. for methodological guidance [32]. To reduce the number of animals needed for experimental purposes, multiple measurements were made in each embryo with caution to limit the duration of anesthesia. Pregnant mice were anesthetized using isoflurane (4% induction dose, 2.0–1.5% maintenance dose) and oxygen (1 L/min). The mean anesthesia time increased slightly with gestational age (range 20–45 min/dam) because the scanning time was increased to accommodate the greater number of measurements that could be recorded as the fetuses matured. Measurements were grouped into distinct gestational ages depending on when the measurements could feasibly be determined (Table 1). Decidual thickness (width and length) and gestational sac thickness and length were measured on days E6.5–9.5. Gestational sac thickness and length, placental length and thickness, crown-rump length, occipital snout length, and stomach diameter were measured on days E10.5–18.5. Crown rump length was measured as the maximum distance from the cephalic pole to the caudal pole. Occipital snout length was measured as the distance from the occipital prominence to the mouth.

**Table 1:** High frequency ultrasound of embryonic and fetal measurements in control and circadian disrupted mice were recorded on all animals over the course of pregnancy on days 6.5–18.5 measuring decidual thickness, gestational sac thickness and length, placental thickness and length, crown-rump length, occipital-snout length, and stomach diameter. Because it is not possible to measure each measurement parameter for the entire duration of the pregnancy, we divided the measurements into three time groups. The first set of parameters were measured on days E6.5–9.5. The second set of parameters were measured on days E10.5–13.5. The final measurements were recorded on days E14.5–18.5. We found significant differences in gestational sac thickness, gestational sac length, placental length, placental thickness, and stomach diameter. All measurements were taken in millimeters, presented as an average ± SD; *p < 0.05.


EXPERIMENTAL GROUP	EMBRYONIC DAY	SET OF PARAMETERS	NUMBER OF EMBRYOS	AVERAGE (MEAN ± SD)

Control	E6.5–9.5	Decidual Thickness (width)	11	2.50 ± 0.65 mm

		Decidual Thickness (length)	11	3.58 ± 1.02 mm

		Gestational Sac Thickness	6	2.44 ± 0.23 mm

		Gestational Sac Length	6	3.85 ± 0.65 mm

Circadian Disrupted	E6.5–9.5	Decidual Thickness (width)	18	2.32 ± 0.62 mm

		Decidual Thickness (length)	18	3.50 ± 1.00 mm

		Gestational Sac Thickness	10	3.41 ± 0.54 mm

		Gestational Sac Length	10	4.61 ± 0.62 mm

Control	E10.5–13.5	Gestational Sac Thickness	25	5.10 ± 1.37 mm

		Gestational Sac Length	25	8.84 ± 2.23 mm

		Placental Length	11	5.12 ± 1.09 mm

		Placental Thickness	11	1.29 ± 0.50 mm

		Crown-Rump Length	22	8.12 ± 2.50 mm

		Occipital-Snout Length	14	4.45 ± 0.74 mm

		Stomach Diameter	8	1.34 ± 0.19 mm

Circadian Disrupted	E10.5–13.5	Gestational Sac Thickness	28	5.33 ± 0.85 mm

		Gestational Sac Length	28	8.87 ± 7.80 mm

		Placental Length	21	5.75 ± 0.95 mm

		Placental Thickness	21	1.68 ± 0.57 mm

		Crown-Rump Length	29	7.36 ± 2.22 mm

		Occipital-Snout Length	24	4.44 ± 0.78 mm

		Stomach Diameter	9	2.00 ± 0.72 mm

Control	E14.5–18.5	Gestational Sac Thickness	18	6.28 ± 1.01 mm

		Gestational Sac Length	18	11.62 ± 1.15 mm

		Placental Length	7	6.56 ± 1.25 mm

		Placental Thickness	7	1.40 ± 0.64 mm

		Crown-Rump Length	17	10.93 ± 1.38 mm

		Occipital-Snout Length	12	5.18 ± 0.65 mm

		Stomach Diameter	10	2.15 ± 0.68 mm

Circadian Disrupted	E14.5–18.5	Gestational Sac Thickness	12	6.81 ± 1.12 mm

		Gestational Sac Length	12	12.52 ± 0.99 mm

		Placental Length	6	8.82 ± 1.56 mm*

		Placental Thickness	6	2.22 ± 0.56 mm*

		Crown-Rump Length	17	11.32 ± 1.37 mm

		Occipital-Snout Length	14	5.74 ± 1.10 mm

		Stomach Diameter	6	3.19 ± 0.84 mm*


### Statistical Analysis

Data are presented as mean ± standard deviation of the mean and analyzed by a two-way ANOVA (treatment x time) with Tukey’s post hoc testing for within-group differences in the presence of a significant main effect. Alpha levels were set at 0.05 a priori (JMP v.17.2.0, SAS, Inc, Cary, NC).

## Results

**Figure 2:**
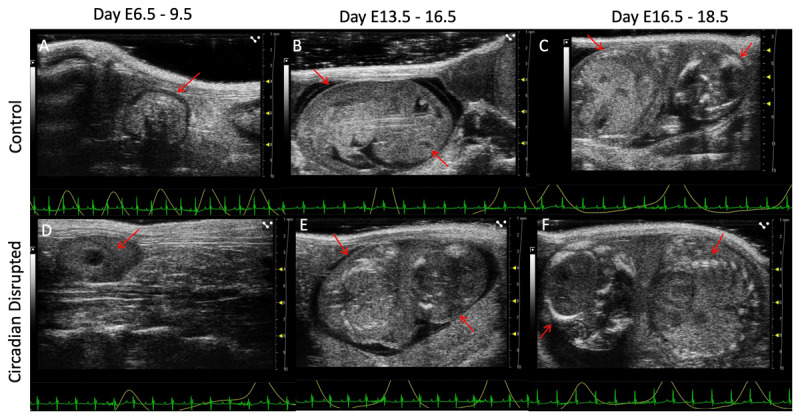
Representative images of high frequency ultrasound images. **A)** Control implantation site at day 8.5. indicated with red arrows; **B)** Control fetus at day 14.5; **C)** Control fetus at day 16.5. Notice the calcified vertebrae and eye indicated with red arrow; **D)** Circadian disrupted implantation site at day 6.5 indicated with red arrow; **E)** Circadian disrupted fetus at day 14.5. **F)** Circadian disrupted fetus at day 17.5 with calcified skull and vertebrae indicated with red arrow.

We used HFUS to monitor and track embryonic and fetal development in control and circadian disrupted mice. Figure 2 shows representative HFUS images illustrating fetal development across gestation. At E6.5–9.5 the embryo was visualized inside the decidua with red arrows pointing to the embryo in both control and disrupted panels (Figure 2A, D). At day E13.5–16.5, note the calcified vertebrae and eye (Figure 2B, E). At day E16.5–18.5, note the calcified skull and vertebrae (Figure 2C, F).

Table 1 summarizes our results broken into experimental groups (control vs. circadian disrupted), the specific embryonic day measured divided into three specific time points E6.5–9.5, E10.5–13.5, and 14.5–18.5, the set of parameters measured such as gestational sac thickness and placental length to name a few, the number of embryos measured for each parameter, and finally the actual value (mm) of the corresponding measurement conducted. All measurements were taken in millimeters, presented as an average ± SD. In the first set of embryonic day groupings (E6.5–9.5) there was no difference in control vs. circadian disrupted experimental groups for decidual thickness (width or length) or gestational sac (thickness or length). Measurements for decidual thickness width in control and circadian disrupted mice were 2.50 ± 0.65 mm and 2.32 ± 0.62 mm, respectively. Measurements for decidual thickness length in control and circadian disrupted mice were 3.58 ± 1.02 mm and 3.50 ± 1.00 mm, respectively. Measurements for gestational sac thickness in control and circadian disrupted mice were 2.44 ± 0.23 mm and 3.41 ± 0.54 mm. Measurements for gestational sac length in control and circadian disrupted mice were 3.85 ± 0.65 mm and 4.61 ± 0.62 mm, respectively.

Next, for the second set of embryonic day groupings (E10.5–13.5) there was no difference in the control vs. circadian disrupted experimental groups for gestational sac (thickness or length), placental (length or thickness), crown-rump length, occipital-snout length, or stomach diameter. Measurements for gestational sac thickness in control and circadian disrupted mice were 5.10 ± 1.37 mm and 5.33 ± 0.85 mm, respectively. Measurements for gestational sac length in control and circadian disrupted mice were 8.84 ± 2.23 mm and 8.87 ± 2.80 mm, respectively. Measurements for placental length in control and circadian disrupted mice were 5.12 ± 1.09 mm and 5.75 ± 0.95 mm, respectively. Measurements for placental thickness in control and circadian disrupted mice were 1.29 ± 0.50 mm and 1.68 ± 0.57 mm, respectively. Measurements for crown-rump length in control and circadian disrupted mice were 8.12 ± 2.50 mm and 7.36 ± 2.22 mm, respectively. Measurements for occipital-snout length in control and circadian disrupted mice were 4.45 ± 0.74 mm and 4.44 ± 0.78 mm, respectively. Measurements for stomach diameter in control and circadian disrupted mice were 1.34 ± 0.19 mm and 2.00 ± 0.72 mm, respectively.

Finally, for the third set of embryonic day groupings (E14.5–18.5) there was no difference in the control vs. circadian disrupted experimental groups for gestational sac (thickness or length), crown-rump length, or occipital-snout length. Measurements for gestational sac thickness in control and circadian disrupted mice were 6.28 ± 1.01 mm and 6.81 ± 1.12 mm, respectively. Measurements for gestational sac length in control and circadian disrupted mice were 11.62 ± 1.15 mm and 12.52 ± 0.99 mm, respectively. Measurements for crown-rump length in control and circadian disrupted mice were 10.93 ± 1.38 mm and 11.32 ± 1.37 mm, respectively. Measurements for occipital-snout length in control and circadian disrupted mice were 5.18 ± 0.65 mm and 5.74 ± 1.10 mm, respectively. However, we did find differences that were previously not indicated past time groupings. For example, we found differences in placental length (p = 0.00016) and placental thickness (p = 0.0332), as well as stomach diameter (p = 0.0186) at days E14.5–18.5 indicating that circadian disrupted animals exhibited a thicker, longer placenta and a larger stomach diameter compared controls. Measurements for placental length in control and circadian disrupted mice were 6.56 ± 1.25 mm and 8.82 ± 1.56 mm, respectively. Measurements for placental thickness in control and circadian disrupted mice were 1.40 ± 0.64 mm and 2.22 ± 0.56 mm, respectively. Measurements for stomach diameter in control and circadian disrupted mice were 2.15 ± 0.68 mm and 3.19 ± 0.84 mm, respectively. These data can be found in Table 1.

## Discussion

Women of reproductive age worldwide are affected by circadian disruption due to shift work, and the impact of maternal circadian disruption during pregnancy on the development of the fetus is largely unknown. To our knowledge, no studies have assessed embryonic and fetal anatomical measurements in circadian disrupted mice using HFUS. Therefore, we aimed to provide information using HFUS to provide data regarding differences in embryonic and fetal parameters *in utero*. Previous studies have linked *in utero* circadian disruptions to problems that can develop and persist later in life. Such complications include cognitive issues, cancer, diabetes, and cardiovascular disease [33]. Yet, no studies have assessed anatomical structures of the developing embryo and fetus perhaps to get a baseline to assess why these issues appear later in life. The current study found differences in developmental parameters of the fetus during days E14.5–18.5. Specifically, placental thickness and stomach diameter are larger in circadian disrupted animals compared to controls. Perhaps one of the most significant findings in our study is the discrepancy in placental thickness between the two animal groups. Placental health has been identified as a marker of fetal health and has been positively correlated with neonatal birthweight [34,35]. Placental thickness, however, has been tied to several placental pathologies including preeclampsia as well as chromosomal abnormalities and anemia. Though not diagnostic, an increased placental thickness does pose an increased risk of adverse perinatal outcomes including intrauterine growth restriction [36,37]. Our data shows circadian disrupted pregnant dams showed a significant increase in placental thickness. Placental thickness has been associated with many maternal and fetal abnormalities like preeclampsia and stunted fetal growth [38]. Pathologic studies on fetal growth restriction have shown associated low placental weight and volume while also having a thicker/globular shape. The pathophysiology being that these placentae have increased apoptosis within the intramural and endovascular trophoblasts [39]. Distal villous hypoplasia is a result of this increased apoptosis and plays a significant role in maternal-fetal gas-exchange secondary to arrested angiogenesis [40,41]. Part of perinatal anatomy screening includes a detailed assessment of the digestive tract to diagnose congenital anomalies. Fetal gastric area has been shown as a useful measurement in the assessment of gastric anomalies. Several case reports have identified large fetal stomach on ultrasound as being associated with gastrointestinal obstruction.

Circadian coordination of clocks in the pituitary, ovary, uterus, and oviduct have distinct, functional molecular clocks that contribute to the timing of events in reproductive physiology [42], yet the regulation and function of these clocks during pregnancy remains largely unknown [43]. The current study only assessed measurements of anatomical structures of the embryo, fetus, and placenta. This study provides no data on long-term effects of the offspring born to circadian disrupted dams. Many questions remain about how circadian disruption during fetal development leads to deleterious consequences later in life. Further research is needed to understand the formation of neurons, the development of anatomical structures, and hormonal programming across the lifespan. Continued research should dive deeper into more molecular, cellular, and subcellular components involved in both circadian rhythm, reproduction, and development.
